# Towards Developing Drought-smart Soybeans

**DOI:** 10.3389/fpls.2021.750664

**Published:** 2021-10-06

**Authors:** Hina Arya, Mohan B. Singh, Prem L. Bhalla

**Affiliations:** Plant Molecular Biology and Biotechnology Laboratory, Faculty of Veterinary and Agricultural Sciences, The University of Melbourne, Parkville, VIC, Australia

**Keywords:** soybean, legume, water stress, abiotic stress, drought, drought-tolerant soybean

## Abstract

Drought is one of the significant abiotic stresses threatening crop production worldwide. Soybean is a major legume crop with immense economic significance, but its production is highly dependent on optimum rainfall or abundant irrigation. Also, in dry periods, it may require supplemental irrigation for drought-susceptible soybean varieties. The effects of drought stress on soybean including osmotic adjustments, growth morphology and yield loss have been well studied. In addition, drought-resistant soybean cultivars have been investigated for revealing the mechanisms of tolerance and survival. Advanced high-throughput technologies have yielded remarkable phenotypic and genetic information for producing drought-tolerant soybean cultivars, either through molecular breeding or transgenic approaches. Further, transcriptomics and functional genomics have led to the characterisation of new genes or gene families controlling drought response. Interestingly, genetically modified drought-smart soybeans are just beginning to be released for field applications cultivation. In this review, we focus on breeding and genetic engineering approaches that have successfully led to the development of drought-tolerant soybeans for commercial use.

## Introduction

Soybean, an important legume, is one of the most widely grown food crops in the world due to its valuable seed composition. In 2019, the annual global soybean production was estimated to be above 333 million tones (Faostat, 2019). Soybean provides an inexpensive source of protein and fats, and natural nitrogen fertilisation for the soil (Foyer et al., 2016). Interestingly, the economic benefits derived from soybean cultivation are not just limited to the food supply; it is also an important industrial crop utilised in producing edible oils, wax, paints, dyes and fibre (Rezaei et al., 2002; Raghuvanshi and Bisht, 2010). Also, meat substitutes based on soybean are extensively used by vegan and vegetarian consumers (Messina and Messina, 2010; Raghuvanshi and Bisht, 2010; Tang, 2017).

Soybean is grown mainly in tropical, subtropical and temperate regions (Fao, 2021). It is a water-intensive crop, requiring substantial water to grow and reproduce (Bhardwaj, 1986). Consequently, rising global temperatures and changing precipitation patterns pose a significant threat to soybean production, especially in under-irrigated or rainfed areas (Jin et al., 2017; Cotrim et al., 2021). It is known that under dry conditions or drought, soybean yield can reduce by more than 50%, causing substantial financial losses to farmers and growers (Wei et al., 2018). Hence, drought is a significant climatic risk that calls for effective mitigation strategies to sustain the supply of soybeans worldwide.

Soybean varieties are classified into maturity groups according to their response to the photoperiod. Early maturing varieties belong to groups 0 to 3, whereas late-maturing varieties fall in groups 6 and onwards (Zhang et al., 2007; Yang et al., 2019). Drought impacts soybeans cultivars differently, as some cultivars are more susceptible than others (Oya et al., 2004; Maleki et al., 2013; Du et al., 2020; Dayoub et al., 2021). Also, the timing of drought stress, whether at the vegetative or the reproductive phase, is important in determining yield loss. Desclaux et al. (2000) investigated the drought-induced phenotypes of early maturity soybean varieties grown in France. They reported that drought stress at vegetative stages led to reduced plant height and a decline in seed number in the early reproductive stages and reduced seed weight in late reproductive stages. The water scarcity between flowering and early seed filling stages can affect branches’ vegetative growth, resulting in decreased branch seed number and reduced branch seed yield (Frederick et al., 2001). A report on the effect of drought on soybeans grown in the semi-arid and semi-humid regions of Huaibei regions of China reported a 73–82% decline in yield when drought stress was applied at flowering and seed filling stages (Wei et al., 2018). Recently, Du et al. (2020) reported that long-term drought stress in reproductive stages decreases biomass allocation to reproductive organs, thereby reducing seed weight in soybean. In addition, drought also impacts the symbiotic nitrogen-fixing ability of soybeans by disturbing nitrogenase activity, which can cause carbon shortage and oxygen limitation leading to poor growth and yield (Arrese-Igor et al., 2011; Collier and Tegeder, 2012; Kunert et al., 2016).

Plants use diverse mechanisms to overcome the adverse effects of drought, and the ability of crops to adjust using adaptive traits is termed ‘drought tolerance’ (Basu et al., 2016). Decreased stomatal opening associated with reduced photosynthesis is a typical drought response observed in plants (Liu et al., 2003; Mak et al., 2014). Abscisic acid (ABA) plays a vital role in reducing water loss under dry conditions (Wilkinson and Davies, 2002; Kim, 2014; Wang et al., 2016). Further drought-tolerant soybeans exhibit higher ABA levels than drought-susceptible varieties (Mutava et al., 2015). ABA, synthesised in plant roots, is transported to the guard cells of leaves, where it induces closure of stomatal openings to reduce water loss (Wilkinson and Davies, 2002). Further, there is evidence that ABA synthesised in leaf xylem also contributes to this process (Malcheska et al., 2017). However, reduction in the stomatal opening leads to reduced CO_2_ assimilation and photosynthesis, affecting growth and development (Cornic and Briantais, 1991; Ohashi et al., 2006; Mutava et al., 2015; Cohen et al., 2021).

**Figure 1:**
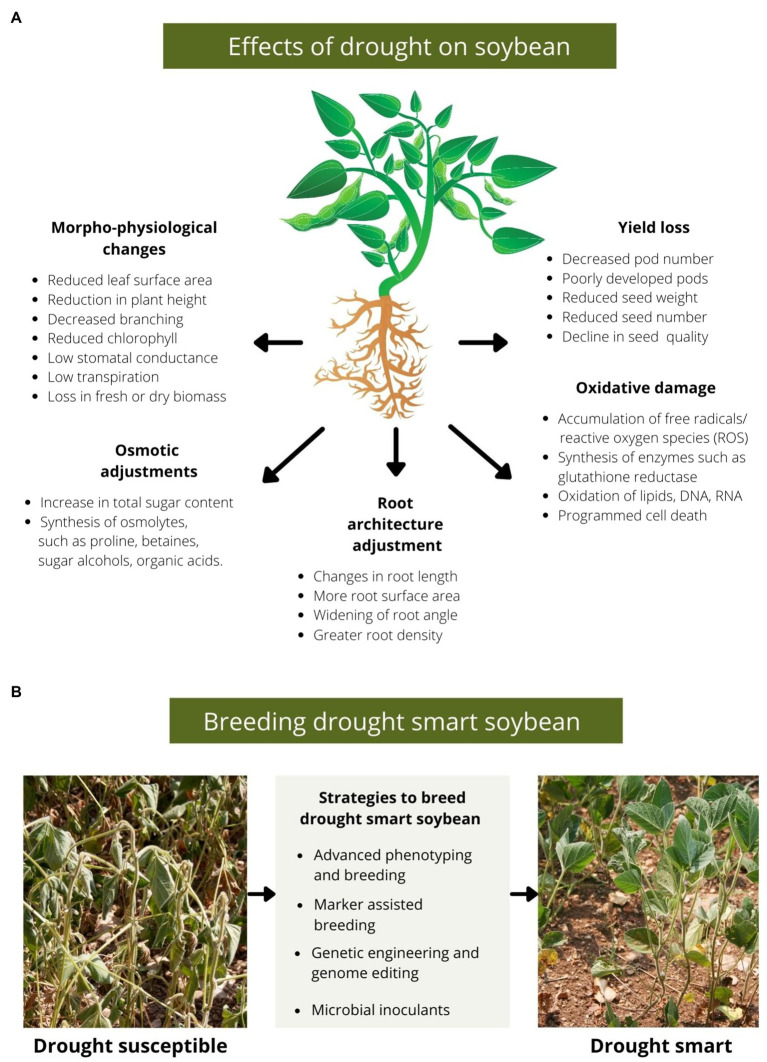
**(A)** A diagram depicting various effects of drought on soybean. Drought induces morphological changes, such as loss in vegetative biomass, accompanied by a reduction in pod number, seed number, seed weight and altered biochemical composition of seeds. Further, cells regulate the effect of drought, synthesising osmolytes, such as proline and sugar alcohols, to balance the osmotic potential for maintaining cell membrane integrity. Also, in many drought-resistant soybean cultivars, roots adjust their architecture in response to water-deficit conditions by changing root length, branching and other phenotypes to absorb more soil moisture. Further, severe drought stress leads to ROS accumulation which can cause cell and tissue damage by oxidising biomolecules. **(B)** An outline of strategies to breed drought-smart soybeans.

Maintenance of cell turgidity is another essential adjustment to survive drought. Under dehydration conditions, cells induce biochemical changes by synthesising necessary metabolites called osmoprotectants (Silvente et al., 2012). These include soluble and complex sugars, sugar alcohols, organic acids and free amino acids (Ashraf and Iram, 2005; Silvente et al., 2012; Kido et al., 2013). Osmoprotectant accumulation in the cell balances the osmotic difference between cell exteriors and the cytosol help to retain water and maintain the integrity of the cell membrane (Yancey, 2005; Basu et al., 2016). An increase in soluble sugars, such as sucrose and fructose, improves homeostasis under stressed conditions. Further, soluble sugars are required for enhanced carbohydrate metabolism, signal transduction and synthesising enzymes and hormones needed to survive under drought (Gupta and Kaur, 2005; Mak et al., 2014; Du et al., 2020). Metabolite profiling of biochemical compounds synthesised during drought stress revealed an increase in pinitol in the leaves of a drought-susceptible cultivar (Silvente et al., 2012). Pinitol is a common sugar alcohol that acts as an osmoprotectant in legumes (Ford, 1984; Streeter et al., 2001; Dumschott et al., 2019). Further, Silvente et al. (2012) reported increased levels of amino acids, such as proline, during drought stress in flowering stages. Proline helps retain water by adjusting the intracellular osmotic potential of the cells (Heerden van and Krüger, 2002). In addition, reactive oxygen species (singlet oxygen) produced in stressed cells can cause severe oxidative damage under prolonged drought conditions. Alia et al. (2001) suggested that proline acts as a scavenger of singlet oxygen. However, the role of proline in quenching singlet oxygen in stressed plants remains debated as Signorelli et al. (2013) demonstrated that proline could not quench singlet oxygen in an aqueous buffer. Figure 1A summarises the effects of drought on soybean.

On the cellular level, largely overlapping signalling mechanisms and phytohormone cross-talks mediate drought response in plants (Basu et al., 2016). With the advent of high-throughput sequencing techniques, several genes or gene families have been identified and characterised in soybean (Wong et al., 2013; Zhang et al., 2020, 2021). Among these, the CUC (NAC; Hussain et al., 2017), MYB (Chen L. et al., 2021), WRKY (Shi et al., 2018), ABA-responsive element binding (AREB; Fuganti-Pagliarini et al., 2017) and dehydration response element-binding proteins (DREB; Nguyen et al., 2019) transcription factor families are some of the prime regulators that control drought response by regulating the synthesis of drought-responsive hormones, such as ABA, ethylene and other drought signalling compounds including Brassinosteroids (Nguyen et al., 2019; Chen L. et al., 2021).

Comparative transcriptomics have further elucidated the molecular mechanisms underlying drought response in soybean (Ha et al., 2015; Hussain et al., 2017). For example, Hussain et al. (2017) identified 28 drought-responsive *GmNAC* genes in soybean and reported that only eight *GmNAC* genes showed high expression levels in drought-tolerant soybean variety; with drought-sensitive cultivars exhibiting lower expression levels. WRKY transcription factors have been highlighted to play vital roles in plant abiotic stress tolerance (Ning et al., 2017; Shi et al., 2018). Shi et al. (2018) identified a drought-responsive soybean WRKY gene, *GmWRKY12*, whose over-expression in a transgenic hairy root assay led to increased proline levels under drought stress. Further, Wei et al. (2019) characterised another *WRKY* gene, *GmWRKY54*, which mediates drought tolerance *via* ABA and Ca^+2^ signalling pathways. The over-expression of GmWRKY54 conferred drought tolerance in soybean (Wei et al., 2019). Other recently identified gene families participating in soybean drought response are *AT-hook motif* (Wang et al., 2021b), *P-type ATPases* (Zhao et al., 2021), *CCT* family (Mengarelli and Zanor, 2021) and *GRAS* (Wang et al., 2020). Identifying new drought-responsive genes is vital for developing drought-smart soybeans. The term ‘drought-smart’ refers to soybean cultivars that can adapt to diverse types of drought, such as early drought, middle-stage drought, later-drought and seasonal drought, by combining different mechanisms of drought resistance, such as drought avoidance, drought tolerance and drought recovery. Drought avoidance is generally achieved by retaining water in plant tissues, either by restricting water loss or using water judiciously to support different plant functions. However, drought tolerance is achieved using adaptive traits, such as biochemical adjustments to maintain cell turgidity and minimise photosynthetic damage (Basu et al., 2016). Further, cultivars with drought avoidance and drought tolerance features can recover fast upon rehydration after a short-term or seasonal drought (Dong et al., 2019). Hence, smart soybeans are able to produce a stable yield in drought-prone areas. Traits, such as improved water retention, restricted transpiration and stable photosynthesis, are desirable to sustain biomass and yield under water-deficit conditions (Wei et al., 2019; Kunert and Vorster, 2020; Yang et al., 2020). A combination of such traits would make a cultivar suitable for cultivation in arid or semi-arid regions (Liu et al., 2005; Abdel-Haleem et al., 2012; Carter et al., 2016). Also, as genome sequencing of soybean cultivars gains momentum, the new genetic diversity resources will aid in developing drought-smart varieties using advanced breeding or genetic engineering methods (Fuganti-Pagliarini et al., 2017; Golicz et al., 2018; Kajiya-Kanegae et al., 2021). Here, we review genetic improvement approaches for improving drought tolerance in soybean.

## Breeding for Drought-Smart Soybean

A combination of advanced phenotyping, molecular breeding and genetic engineering approaches can be employed to breed drought-smart soybeans (Figure 1B). Using conventional breeding techniques, pre-selected soybean germplasms (donor cultivars) with desired drought-responsive traits can be crossed to introduce favourable alleles in the resulting populations. However, extensive screening of subsequent generations is required to select a line with stable characteristics for continued cultivation (Carter et al., 2016). Further, genetic transformation offers the possibility of targeted gene expression under constitutive or inducible promoters (Ribichich et al., 2020). For example, *Arabidopsis thaliana AtMYB44* gene was transformed in soybean using *Agrobacterium-*mediated transformation which resulted in improved soybeans with better yield under water-deficit conditions in the field (Seo et al., 2012). Improvements in tissue culture regeneration of commercial soybean cultivars and optimization of *Agrobacterium-*mediated transformation methods will also facilitate engineering soybeans for drought tolerance (Raza et al., 2017, 2019). Recently, more precise gene editing technique, CRISPR/cas9, has shown promising results in modifying soybean’s genome in a targetted manner to obtain more specific gene modifications. CRISPR/cas9 has been successfully employed to characterise soybean drought-responsive genes using knock-down approaches. For example, CRISPR/cas9-mediated mutagenesis of soybean circadian rhythm genes *(GmLCLs)* generated mutant plants with decreased water loss under dehydration stress conditions (Yuan et al., 2021). Interestingly, breeding and genetic transformation methods have successfully delivered improved soybeans that have been tested in laboratory, glasshouse or field conditions and a few of these varieties have also been approved for commercial production. In the sections below, we review recent examples of soybeans improved for drought tolerance *via* breeding or transgenic approaches.

### Breeding and High-Throughput Phenotyping

Molecular breeding approaches for enhancing drought tolerance, such as marker-assisted selection, quantitative trait loci (QTL) mapping, GWAS and genomic selection, depend upon the extent of existing genetic diversity for the desirable traits. High genetic diversity of soybean makes it feasible to select cultivars with drought tolerance properties (Kajiya-Kanegae et al., 2021). Some of the traits to address water deficit are slow canopy wilting, water-conserving transpiration response, dense root surface area and low stomatal conductance (Kim, 2014; Basu et al., 2016).

Using phenotypic trait selection approaches, a high-yield and drought-tolerant cultivar was bred by the US Department of Agriculture and North Carolina State University researchers. This study involved extensive screening of soybean germplasm collected from around the world. Fifteen years of rigorous selection led to discovering a slow-wilting landrace PI 416937 from Japan and another resistant cultivar from Nepal, PI 471938. Continuous breeding efforts led to the development of a new drought-tolerant soybean variety released as ‘USDA-N8002’ (maturity group VIII) for field use (Carter et al., 2016).

Recently, high-throughput phenotyping of plant populations has widened trait identification by reducing the timeline and physical labour involved in the manual screening processes (Crusiol et al., 2021; Zhou et al., 2021). For example, remote sensing has shown great potential in analysing the genotypes of plants in a non-destructive way. These remote sensing mechanisms of phenotyping involve thermal, spectral and hyperspectral imaging techniques (Li et al., 2014). A simple example of spectral measurement is the estimation of chlorophyll content using the soil plant analysis development metre which measures red *vs.* infra-red light (Yuan et al., 2016). Hyperspectral imaging is an advanced imaging method used to detect material across the entire electromagnetic spectrum. It works on the principle that certain elements have unique spectral fingerprints that can be used to identify materials by analysing the image of the scanned object (Crusiol et al., 2021).

Crusiol et al. (2021) employed a leaf-based hyperspectral reflectance method to distinguish soybean genotypes under different moisture conditions and at different phenological stages. Leaf-based hyperspectral reflectance was collected from soybean genotypes growing under different watering conditions over multiple cropping seasons. Short-wave infra-red wavelength (1300–2,500nm) was critical in these measurements, as it can effectively detect vegetation water status. Principal component analysis of spectral datasets of soybean genotypes showed 94% variance in the first three components, indicating that spectral data could successfully distinguish the soybean genotypes.

Multispectral and visible RGB camera imaging was also performed by Zhou et al. (2021) to estimate the yield of soybean genotypes under drought stress. They employed the unmanned aerial vehicle technique for collecting image data to develop a method of yield estimation for large breeding populations. Images of 972 breeding lines were captured at vegetative (R6), early and late reproductive phases (R1 and R6-R8). By assessing the image features related to plant height, canopy colour and canopy texture, they built a deep learning model which could explain the yield to up to 78%. The yield of slow- and fast-wilting plants belonging to three genotypes (maturity group 3, 4 and 5) was assessed, and it was found that the fast-wilting group produced less average yield (986.5kg/ha) as compared to the slow-wilting group (1,395kg/ha). Hence, high-throughput imaging for phenotyping has a remarkable potential for assessing large populations to identify plants with drought-tolerant traits.

### Marker-Assisted Breeding

Marker-assisted breeding is another promising approach for developing drought-tolerant soybeans. It relies on identifying variations in chromosomal regions, known as QTLs. QTLs that make a genotype more robust than others for drought tolerance are particularly valuable for breeding. Marker-assisted breeding uses DNA markers linked to specific QTLs for selecting genotypes with the desired alleles (Zhang et al., 2015). For example, Chen H. et al. (2021) identified QTLs related to primary root length on chromosome 16 of soybean. This QTL accounts for 30.25% variation in phenotype and will assist in developing of markers for root-length selection, which is an important trait for drought tolerance. Dhungana et al. (2021) identified QTLs associated with flooding stress at the V1-V2 stage of soybean. They analysed a recombinant inbred line (RIL) population derived from crossing a drought-susceptible (NTS116) and drought-tolerant (Danbaekkong) soybean cultivar. Using a composite interval mapping technique, they identified 10 QTLs related to flood tolerance at the V1-V2 stage of soybean. These QTLs can cause up to 30.7% phenotypic variations and can be useful for future soybean improvement programmes. Table 1 shows a list of major QTLs associated with soybean drought response. Further, other QTLs identified with soybean drought tolerance can be found at www.soybase.org, one of the prime repositories of soybean genetic resources.

**Table 1:** A summary of QTLs identified with soybean drought tolerance.

Trait	Number of QTLs	QTL name and *G. max* (Gm) chromosome number	Donor line	Reference
Slow canopy wilting	2	qSW (Gm06), qSW (Gm10)	Magellan PI 567731	Ye et al., 2019
Slow canopy wilting	7	qSW (Gm02), qSW (Gm04), qSW (Gm05), qSW (Gm12), qSW (Gm14), qSW (Gm17), qSW (Gm19)	PI416937 Benning	Abdel-Haleem et al., 2012
Basal root thickness (BRT), Lateral root number (LRN), Maximum root length (MRL), Root fresh weight (RFW), Root dry weight (RDW), Shoot fresh weight (SFW), Shoot dry weight (SDW), and Ratio of SFW/SDW	12	qBRT001 (Gm18), qLRN001 (Gm18), qMRL001(Gm06), qMRL002 (Gm06), qMRL003 (Gm03), qRFW001 (Gm08), qRFW002 (Gm08), qRDW001 (Gm08), qRDW002 (Gm03), qSFW001 (Gm08), qSFW002 (Gm06), qSFW003 (Gm01), qSFW004(Gm03), qSDW001 (Gm08), qSDW002 (Gm18), qSDW003 (Gm03), qSFW/SDW001 (Gm14), SFW/SDW002 (Gm06), qSFW/SDW003 (Gm13)	Essex Forrest	Williams et al., 2012
Canopy wilting	4	QTL-Molecular Linkage Group (MLG) A2 (Gm08), QTL-MLG B2 (Gm14), QTL-MLG D2 (Gm17), QTL-MLGs F (Gm13)	KS4895 Jackson	Charlson et al., 2009
Drought Susceptibility Index in the field (DSI-F) and Drought Susceptibility Index in the glass house (DSI-G)	10	DSI-F (Gm01), DSI-F (Gm06), DSI-F (Gm07), DSI-F (Gm12), DSI-F (Gm16), DSI-F (Gm20), DSI-G (Gm05), DSI-G (Gm05), DSI-G (Gm17), DSI-G (Gm17)	Kefeng 1 Nannong1128-2	Du et al., 2009
Water use efficiency (WUE)	1	cr497-1 (Gm16), K375-ln (Gm16), B031-ln (Gm18), A089-1(Gm12)	PI416937 Young	Mian et al., 1996

### Genetic Engineering

Genetic engineering approaches offer viable opportunities for accelerated crop improvement (Khan et al., 2020; Lohani et al., 2020; Arya et al., 2021). Genes controlling traits, such as flowering time, disease resistance and lipid profile, have been identified and used as targets for soybean improvement (Haun et al., 2014; Arya et al., 2018; Ngaki et al., 2021). Similarly, high-throughput genome and transcriptome sequencing have led to identification of key transcriptional regulators of soybean drought response. Among these, the DREB (Nguyen et al., 2019; Zhou et al., 2020), AREB (Fuganti-Pagliarini et al., 2017), NAC (Hussain et al., 2017; Yang et al., 2020), MYB (Chen L. et al., 2021) and WRKY (Ning et al., 2017; Shi et al., 2018; Wei et al., 2019) are the prime transcription factor families mediating abiotic stress responses. Gain of function and gene-knock-down approaches, such as RNAi and CRISPR/cas9, have yielded valuable information on how complex gene networks regulate dehydration stress physiology in soybean. For example, Yang et al. (2020) characterised GmNAC8 transcription factor as a positive regulator of soybean drought stress. NAC transcription factor family is primarily involved in plant growth and stress response. *GmNAC8* was cloned under the control of 35s promoter for gene over-expression, and CRISPR/Cas9 was used to knock down *GmNAC8*. The performance of over-expression and knock-down lines was analysed under drought stress by withholding water supply for 14days. Interestingly, *GmNAC8* over-expression lines had significantly high superoxide dismutase levels and proline content, which are both indicators of drought tolerance in plants. Further, as the water supply was restored, the recovery rate of over-expression lines was relatively high (up to 96%) compared to WT lines which showed only a 40% recovery rate. Interestingly, the *GmNAC8* knock-down lines only had a 5 to 14% recovery rate.

MYB transcription factors regulate the biosynthesis of secondary metabolites for stress responses. Recently, Chen H. et al. (2021) characterised GmMYB14, which participates in drought tolerance and high-density soybean yield by affecting plant architecture through the Brassinosteroid pathway. *GmMYB14* over-expression lines were compact with decreased plant height, internodal length, leaf surface area and petiole angle. However, the transgenic plant showed an increase in node number on the main stem and increased branch number, which contributed to enhanced yield under high-density cropping (20cm intervals) conditions. Further, under drought stress, pod number, seed number and seed weight per plant were significantly improved in soybeans over-expressing *GmMYB14* compared to WT. Hence, as we face the challenge of producing more food from our limited arable land, plants that can withstand drier conditions and generate better yield under high-density cropping have the potential of ensuring future food security. Table 2 shows a list of genes or gene families recently identified to soybean drought response.

**Table 2:** A summary of recently characterised gene targets for engineering drought tolerance in soybean.

Genes	Method used to identify genes	Outcome	Reference
*LHY1a* and *LHY1b*	CRISPR/cas9 to generate LHY mutants and transgenic hairy root assay to characterise *LHY1a* and *LHY1b*	*LHY1a* and *LHY1b* are negative regulators of drought tolerance as *LHY* mutants showed improved drought tolerance. Also, *LHY1a* and *LHY1b* regulate drought tolerance *via* ABA pathways.	Wang et al., 2021a
*GmTGA15*	Transgenic hairy root assay to over-express *GmTGA15*	Transgenic lines showed improved chlorophyll and proline content under drought	Chen, Z et al., 2021
*GmNTF2B-1*	Transgenic hairy root assay to over-express *GmNTF2B-1*	*GmNTF2B-1* improves ROS scavenging under drought stress	Chen, K et al., 2021
*GmDUFF428-70*	Transgenic hairy root assay to over-express *GmDUFF428-70*	Over-expression lines showed less wilting of leaves, increased chlorophyll, proline and relative water content.	Leng et al., 2021
*GmLCL*	CRISPR/cas9 to generate quadruple LCL mutants	Mutants had decreased water loss under water-deficit conditions. *GmLCL* genes are negative regulators of ABA signalling.	Yuan et al., 2021
*DREB* genes (76 targets were identified)	Genome-wide analysis and transcriptome analysis	Two soybean homologues of Arabidopsis *DREB3* and *ERF039*were highly expressed in early drought stages. Homologues of Arabidopsis *RAP2* genes were highly expressed in late drought stages	Zhou et al., 2020
*GmGRAS37*	Genome-wide identification of *GmGRAS* family and hairy root assay to characterise *GmGRAS37* under drought stress.	Soybean hair roots over-expressing *GmGRAS37* showed improved tolerance to drought	Wang et al., 2020
*GmWRKY54*	Gene over-expression under constitutive and drought-induced promoter	Enhanced stomatal closure to reduce water loss was observed in transgenic lines. ABA and Ca2+ signalling activated.	Wei et al., 2019
*GmDREB6*	Gene over-expression	Enhanced proline accumulation was observed in transgenic lines	Nguyen et al., 2019
*GmWRKY12*	RNA seq and quantitative PCR	Transgenic hairy roots assay confirmed that *GmWRKY12* improves drought tolerance in soybean	Shi et al., 2018
*GmNAC* (28 targets were analysed)	Genome-wide identification and quantitative PCR	Eight genes (*GmNAC004, GmNAC021, GmNAC065, GmNAC066, GmNAC073, GmNAC082, GmNAC083 and GmNAC087*) were highly expressed in drought-resistant cultivars	Hussain et al., 2017

Dehydration responsive element binding (DREB) and AREB transcription factor family genes are known to mediate drought inducible gene expression (Fuganti-Pagliarini et al., 2017; Zhou et al., 2020). The transgenic soybean lines over-expressing transcription factors DREB1A, DREB2A and AREB1 have been field tested for the agronomic and physiological performance under-irrigated and non-irrigated conditions (Fuganti-Pagliarini et al., 2017). Improved water use efficiency and leaf area index were reported in *35S: AtAREB1FL* lines compared to the control lines. Also, *35S: AtAREB1FL* lines had the highest yield under non-irrigated conditions, similar to the yield of *35S: AtAREB1FL* lines under-irrigated conditions. Interestingly, in non-irrigated conditions, the oil and protein contents of seeds were not affected by the insertion of DREB1A or DREB2A or AREB1 transcription factors.

Recently, soybeans expressing the sunflower (*Helianthus annus*) transcription factor, HaHB4, were approved for production by the US Department of Agriculture. *HaHB4* is a water-deficit responsive sunflower transcription factor whose over-expression in Arabidopsis led to improved drought tolerance (Manavella et al., 2008). *HaHB4* was cloned under constitutive *35S* promoter and inducible *HaHB4* promoter. Soybeans expressing HaHB4 were studied under glasshouse and field conditions by Ribichich et al. (2020). Transgenic lines designated as b10H performed best under field trials when genotype to environment interaction (G×E) was analysed. The b10H soybeans had better yield (seed number) under warm and dry conditions, not compensated by a decrease in seed weight. Under water-deficit conditions, b10H produced 43.4% more yield compared to WT (Williams 82). Also, the diameter of epicotyls, internode and xylem was wider in b10H soybeans as compared to WT plants. Further, b10H plants had a significantly high photosynthetic rate (at R5 and R6 stage) under warm field conditions. Molecular analysis showed that transcripts of heat shock proteins homologous to *Arabidopsis thaliana HSC70-1(At5G02500), HSFB2A (At5G62020), Hsp81.4(At5G56000)* and *HOT5 (At5G43940)* were differentially regulated in soybeans expressing HaHB4 (Ribichich et al., 2020). Due to its exceptional field performance, the trait HaHB4 has received regulatory approvals in Argentina, Brazil, Paraguay and Canada (Businesswire, 2021).

### Microbial Inoculants for Drought-smart Soybean

Symbiotic rhizobium species associated with soybean root nodules benefit plant growth *via* mediating biological N fixation (Jaiswal et al., 2021). Reduced photosynthesis under drought conditions disturbs oxygen balance in nodules triggering premature nodule senescence (Arrese-Igor et al., 2011; Kunert et al., 2016). Symbiotic association of soybean plants with arbuscular mycorrhizal fungi, *Glomus mosseae and G. intraradices,* has been reported to alleviate drought-induced nodule senescence (Porcel and Ruiz-Lozano, 2004; Takács et al., 2018). Further, it has been demonstrated that extenuation of premature nodule senescence is mediated by the induction of high glutathione reductase in soybean roots and nodules. Glutathione reductase activity likely exerts its influence by reducing oxidative damage to biomolecules (Porcel and Ruiz-Lozano, 2004; Prabha and Sharadamma, 2019; Meena et al., 2021). Hence, arbuscular mycorrhizal fungi can play important roles in alleviating the impact of drought at root-nodule interfaces in soybeans. Recently, co-inoculation of rhizobia and mycorrhizal fungi has shown enhanced soybean tolerance to drought stress providing a cost-effective strategy for improving soybean productivity (Igiehon et al., 2021).

## Conclusion

Soybean is a crop of immense economic importance (Johnson and Myers, 1995; Messina and Messina, 2010). Vast genetic diversity has been reported in soybean germplasm, and the increasing availability of soybean genetic resources has instigated the development of drought-smart soybeans (Carter et al., 2016; Ribichich et al., 2020; Kajiya-Kanegae et al., 2021; Chen L. et al., 2021). Modern breeding and advanced biotechnology methods have shown promising results, and market-ready drought-tolerant soybeans have been released in some parts of the world (Carter et al., 2016; Ribichich et al., 2020). However, soybean production is still dependent on adequate irrigation facilities in many regions, especially in under-developed and developing nations (Droppers et al., 2021; Suriadi et al., 2021). As genetic and non-genetic improvement methods are tested on more cultivars, the dependency of soybean production on rainfall or heavy irrigation should reduce. With changing precipitation patterns and a hotter climate, drought-tolerant soybeans will play a significant role in ensuring our future food security.

## Author Contributions

All authors contributed to the article and approved the submitted version.

## Conflict of Interest

The authors declare that the research was conducted in the absence of any commercial or financial relationships that could be construed as a potential conflict of interest.

## Publisher’s Note

All claims expressed in this article are solely those of the authors and do not necessarily represent those of their affiliated organizations, or those of the publisher, the editors and the reviewers. Any product that may be evaluated in this article, or claim that may be made by its manufacturer, is not guaranteed or endorsed by the publisher.
